# The effect of alcohol strength on alcohol consumption: a randomised controlled cross-over pilot trial

**DOI:** 10.1186/s40814-018-0328-y

**Published:** 2018-08-14

**Authors:** Parvati R. Perman-Howe, Emma L. Davies, David R. Foxcroft

**Affiliations:** 1 Oxford Brookes University, Marston Campus, Jack Straw’s Lane, Oxford, OX3 0FL UK; 2 Oxford Brookes University, Gipsy Lane Campus, Gipsy Lane, Oxford, OX3 0BP UK

**Keywords:** Alcohol, Alcohol strength, Public health, Prevention, Intervention, Licenced premises, Pub, Bar, Pilot trial

## Abstract

**Background:**

Effective interventions are required to reduce alcohol consumption and its associated harms at the population level. Reducing the alcohol content of beverages has the potential to reduce alcohol consumption through non-conscious processes. Before implementing a randomised controlled trial (RCT) to assess the effect of alcohol strength on alcohol consumption, its feasibility needs to be established. This study aims to pilot a RCT and obtain data to estimate key parameters required when designing a RCT. These key parameters include the direction and size of the intervention effect, the efficacy and efficiency of the study processes and the rates of licenced premises recruitment, participant recruitment and attrition.

**Methods:**

A double-blind randomised controlled cross-over pilot trial comparing the number of units of reduced strength lager consumed and the number of units of regular strength lager consumed in a single drinking occasion within licenced premises in the UK.

Descriptive statistics will report the efficacy and efficiency of the study processes and the rates of licenced premises recruitment, participant recruitment and attrition. Mean and 95% confidence intervals will be used to compare the consumption of alcohol and the duration of participation in study sessions, between the intervention arm and the control arm. The mean and standard deviation of UK units of alcohol consumed will be used to calculate a sample size for a definitive RCT.

**Discussion:**

This is the first naturalistic experimental study to assess the effect of alcohol strength on alcohol consumption in a single drinking occasion within licenced premises. Results from this pilot study will establish the feasibility of, and inform key data parameters for, a larger scale study.

**Trial registration:**

The trial is registered in the American Economic Association (AEA) Randomised Controlled Trial (RCT) Registry as of 16 June 2017. The unique identifying number is AEARCTR-0002266.

## Background

Excessive alcohol consumption is a causal factor for many chronic health conditions, and it increases the risk of intentional and non-intentional harm through violence and injury [1, 2]. In 2015, there were 8758 avoidable deaths in the United Kingdom (UK) that were directly caused by alcohol [3]. A study with over 55,000 UK participants found that of the 69% who reported drinking alcohol, 27% reported drinking at levels that are classed as high risk [4]. Moreover, 2.5 million people who regularly drink alcohol report exceeding weekly alcohol thresholds in a single drinking occasion [5]. The financial burden of alcohol-related harm is estimated to annually cost UK society betwen 1.3 and 2.7% gross domestic product (GDP) [6].

The most effective alcohol harm prevention interventions may be those that target non-conscious processes and that are readily scalable to the population level [7–11]. These include interventions that alter the properties or placement of external stimuli, such as the strength of alcoholic products [10–12]. Such interventions could be particularly beneficial within licenced premises where individuals may not have direct access to information such as the strength of alcoholic products. For example, lager “taps” often display a brand logo but do not incorporate information about the strength of the product. Labelling drinks as lower in strength has been shown to increase the amount of alcohol consumed within a laboratory setting [13]. However, we propose that when information about alcohol strength is not forthcoming, such as when lager is purchased from the “tap”, most consumers will not consciously seek this information. Therefore, consumers cannot knowingly compensate for drinking lower strength alcohol. Reducing the alcohol content of popular lager products that are sold on “tap”, or in other situations where information about alcohol content is not readily available, may lead to a reduction in alcohol consumption. Interventions that utilise non-conscious processes have the added benefit of potentially reducing health inequalities as their recipients are not required to be health literate and numerate or have high-functioning cognition: lack of which are more prevalent with higher levels of deprivation [9, 14].

Reducing the alcohol content of drinks, thereby reducing the number of alcohol units each drink comprises, was proposed as a means to reduce alcohol consumption by the UK Coalition Government (2010–2015) as part of the Public Health Responsibility Deal (PHRD) [15]. Between 2011 and 2013, 1.3 billion UK units of alcohol were removed from the UK market by reductions in the alcohol content of beverages. This equates to the average strength of beer falling from 4.42 to 4.14% alcohol by volume (ABV) [16]. There is scope to further reduce the ABV of alcohol in the UK market, but to date, there is insufficient evidence that reducing the alcohol content of drinks reduces the number of alcohol units consumed.

There is a paucity of studies that assess the effect of the strength of alcohol on alcohol consumption within a naturalistic setting. Most studies of alcohol strength are strength discrimination studies. The majority of these are laboratory based [17–21] and one study was based within a mocked-up lounge in a community centre [22]. All but one incorporate beer, or beer and spirits, and a single study focuses on wine [20]. These studies all support the hypothesis that people cannot readily distinguish between alcoholic products of different strength, which indicates that there is potential to subconsciously alter alcohol consumption by altering the ABV of alcoholic products. An experiment with Canadian students found that participants reported similar levels of enjoyment and perceived intoxication after consuming an equivalent volume of lower strength lager and regular strength lager [23]. However, this study has numerous limitations: it used a small sample of male students, it was based within a classroom, and participants were restricted to the amount of alcohol they could consume. A more robust study that assessed the effect of the strength of beer and mixed spirit-based drinks on consumption supports the hypothesis that reducing the alcohol content of drinks does not lead to an increase in the volume of alcohol consumed, therefore reducing consumption [24]. There were also limitations in this study’s design, however, most notably that it was based within closed student fraternity parties comprising a single fraternity at one university in the United States of America (USA) [24].

High-quality research is warranted to assess the effect of alcohol strength on consumption within a naturalistic environment. Prior to a definitive randomised controlled trial (RCT), a pilot study is required to test feasibility and estimate key parameters for the RCT’s design. This study aims to determine the feasibility of a RCT, which would assess whether people consume fewer units of Bud Light lager 3.5% ABV (BL) compared to Becks lager 4.8% ABV (B) in a single drinking occasion within licenced premises. The intervention product, BL, is one of few mainstream lagers sold in the UK that is below 3.8% ABV, and it is reported to have retailed well since its UK launch in March 2017 [25]. Results of a taste discrimination experiment to establish a control product for the pilot trial concluded that, out of a range of mainstream regular strength lagers, B tasted the most similar to BL (unpublished observations: Perman-Howe, Davies and Foxcroft). To reduce confounding from difference in taste, BL and B were therefore chosen as the intervention and control products for the pilot trial.

The current study is defined as a randomised pilot trial, in accordance with Eldridge et al.’s conceptual framework for defining feasibility and pilot studies in preparation for a RCT [26]. That is, the future RCT, or parts of it, including the randomisation of participants, will be conducted on a smaller scale to see if it can be done. It could also legitimately be called a randomised feasibility study, but for clarity it will not be referred to as a feasibility study [27]. Additionally, and in line with Teare et al.’s definition of a pilot study, it will provide data with which to estimate key parameters for the design of a RCT [27]. It is an external pilot study: a stand-alone piece of work that has been planned and will be carried out independently to a main study [28].

## Methods/design

### Aim/objectives

The overall aim of the study is to pilot a double-blind randomised controlled cross-over trial to assess the effect of alcohol strength on alcohol consumption. The feasibility objectives are to establish whether:

- Components of the study protocol are efficient and work together or can be amended to be or do so
- A sufficient number of licenced premises can be recruited to host the study
- The participant recruitment rate per study session is sufficient
- Participant retention is sufficient
- The sample size derived from data obtained in the study is achievable for a future definitive trial.

The participant-centred objective is to establish whether:

- Estimations of the mean and 95% confidence intervals of the number of UK units of alcohol consumed by participants in a single drinking occasion support the hypothesis that people consume fewer UK units of alcohol when they consume reduced strength lager.

### Design

The study is a double-blind randomised controlled AB/BA cross-over pilot trial. The AB/BA cross-over design means that each participant experiences both the intervention and the control conditions, within a two-arm trial, on separate occasions and in a randomised order. There will be a four-week washout period between each participant´s study sessions, which is deemed adequate for participants to have desensitised to the sensory aspects of the alcohol they consumed in their first study session. There is no risk of carryover effects from the alcohol consumed during participants’ first study session, as alcohol is expelled from the body at the rate of approximately one unit per hour. There is a possibility of period effects as the characteristics of the participants may alter between their two study sessions: this will be tested for using a *t* test between the sequences AB and BA.

### Setting

The pilot trial will be based within on-trade licenced premises in the South East of England, and London, UK. For more information, refer to the “Recruitment of licenced premises” section.

### Participants

Fifty-two adults who regularly consume lager within licenced premises will be recruited for the pilot trial. See Table 1 for the Standard Protocol Items: Recommendations for Interventional Trials (SPIRIT).

**Table 1:** Standard Protocol Items: Recommendations for Interventional Trials (SPIRIT) figure

Time point	Study period							
	Enrolment	Allocation	Post-allocation					Close-out
	≥ 24 h before study session 1	When participant arrives for study session 1	Study session 1	End of study session 1	When participant arrives for study session 2: 4 weeks after study session 1	Study session 2	End of study session 2	Approx 48 h after the final study session at the venue
Enrolment								
Eligibility screen	X							
Consent		X						
Allocation		X						
Interventions								
Bud Light			X			O		
Becks			O			X		
Assessments								
BrAC reading		X		X	X		X	
Time recorded		X		X	X		X	
UK units of alcohol consumed counted				X			X	
Questionnaire				X			X	
Debrief email/letter								X

Participants must meet all of the inclusion criteria and not meet any of the exclusion criteria (Table 2).

**Table 2:** Inclusion/exclusion criteria

Inclusion	Exclusion
≥ 18 years old	Has ever sought help, or been treated, for an alcohol dependency
Regular drinker of lager within a licenced premises (≥ once in the past three months)	Has an illness or condition with which they should not be consuming alcohol
Able to attend two study sessions	Is on medication with which they should not be consuming alcohol
Provides informed consent	Pregnant
	Has a BAC > 35 μg/100 ml breath when they arrive for a study session

**Fig. 1:**
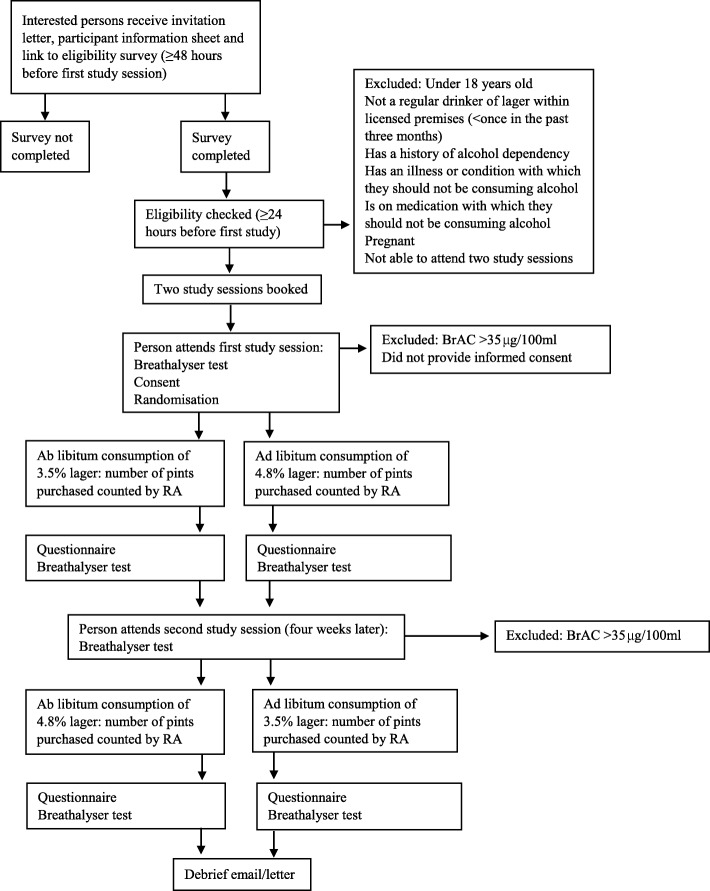
Participants’ pathways

Figure 1 illustrates the participants’ pathways through the pilot trial.

### Interventions

#### Intervention product

The intervention product is Bud Light lager 3.5% ABV. It will be poured from 440-ml cans into a pint glass so that a full pint (568 ml) is served. Participants may consume the intervention product ad libitum during their study sessions.

#### Control product

The control product is Becks lager 4.8% ABV. It will be poured from 440-ml cans into a pint glass so that a full pint (568 ml) is served. Participants may consume the control product ad libitum during their study sessions.

#### Provision of intervention/control products

Before a study session, the principal investigator (PI: PPH) will estimate the number of study-specific drinks that will be required during the forthcoming session and the corresponding number of 440-ml cans of BL and B will be completely de-identified with duct tape.

The intervention and control products will be chilled in a fridge 24 h before a study session.

Participants will pay for study-specific drinks and drink them ad libitum during a study session. The price of the drinks will be specific to each venue and reflect a small reduction (approximately 33%) in the normal price for similar products at each venue: as participants will not be able to choose their brand of lager and will not know what brand they are purchasing.

When a participant wishes to purchase a study-specific drink, they will go to a makeshift bar area that the research assistant (RA: JW) will be manning. The RA will exchange the participant’s money for BL or B and stamp their randomisation card: a coloured square on the randomisation card will refer to a coloured sticker on the lager cans to notify the RA which cans to serve.

The drinks will be poured from 440-ml lager cans into pint glasses so that a full pint is served: each pint will therefore contain more than one can of lager. The RA will be responsible for disposing of the empty cans and giving the used glasses to the bar person (BP) to put into the glass washer. The RA should not have to restock the designated fridge space as the PI will ensure that the fridge space is large enough to accommodate plentiful alcohol for each study session: based on an estimation of each participant drinking four pints.

Participants will be briefed that they may purchase soft drinks from the normal bar area or be given free tap water by the RA, but the only alcoholic drinks they should purchase during a study session are the study-specific lagers.

#### Discontinuing or modifying intervention/control products

The intervention and control products will not be replaced or modified during the pilot trial.

### Outcome measures

The primary outcome is the feasibility of a RCT. A RCT would be deemed feasible if it adheres to the following points:

1. Components of the study protocol are efficient and work together or can be amended to be or do so. These include:
  The administration of data collection tools
  The consent process
  The randomisation process
  Data management processes
  The roles and requirements of study personnel
2. The licenced premise recruitment rate is a minimum of one per month for a minimum of 4 months or until four licenced premises have been recruited.
3. Participant recruitment rate is a minimum of four per study session.
4. The rate of attrition for the pilot trial is less than or equal to 30%, and this does not vary by more than 10% according to the arm of the trial.
5. The sample size is achievable to obtain within a year based on the recruitment rate of licenced premises and participants.

In addition, one participant-centred outcome will be assessed:

1. Whether estimations of the mean and 95% confidence intervals of the number of UK units of alcohol consumed by participants in a single drinking occasion, when they consume BL and B, suggest that people consume fewer UK units of alcohol when they consume reduced strength lager.

### Sample size

As there are no data from previous studies on which to base a statistical calculation, and there is no consensus in the literature about the required sample size for pilot trials, the sample size has been calculated using preliminary datasets. These preliminary datasets are based on the hypothesis that there is no significant difference between the number of alcoholic drinks individuals consume, regardless of their ABV, which has been shown in a previous study [24].

Firstly, preliminary datasets were created for 40 patrons at each of four different licenced premises with different demographics, based on their average patron’s characteristics, i.e. age and gender. The mean patrons’ age was calculated from each licenced premises’ preliminary dataset. Mean age was used to estimate the number of units that each of the 40 hypothetical patrons would consume under normal conditions, based on age-related population data for alcohol consumption [29]. The estimated number of units consumed under normal conditions was reduced by 27% to give the estimated number of units consumed under the intervention: the difference in ABV between BL and B is − 27%. Estimated mean units and SD for units consumed from BL and B were calculated; a conservative estimate for SD in the intervention arm was used: the same SD as in the control arm. Where the licenced premises’ population incorporated a higher proportion of male to female consumers, this was accounted for in the calculations and the mean consumption increased accordingly. From these data, the estimated mean difference and SD of the mean difference of UK units of alcohol consumed were calculated. These data enabled delta to be calculated, 0.3977.

Preliminary data for the licenced premises with the largest SD was used to calculate the sample size using the Rstudio software ‘R Stats Package’ and the function power.t.test [30]: this provided the most conservative calculation of sample size. The figures that were inputted into Rstudio were alpha = 0.05, beta (power) = 0.8, delta = 0.3977, SD = 1. The sample size for a two-sided paired *t* test was calculated as 52: 52 participants participating in two trial arms. As this does not account for attrition, participants who drop out of the pilot trial will be replaced.

#### Trial withdrawal

Participants who wish to withdraw from the study will be directed to contact the PI via email or telephone or express this to the PI verbally during a study session.

Participants who are seen by the PI, the RA or the BP to be obviously and persistently breaching the protocol will be withdrawn from the study. These breaches include consuming any alcohol other than the study-specific lager which they have purchased, supplying non-participants with study-specific drinks, and disposing of any study-specific lager without declaring it.

#### Definition of end of study

The study will officially end when the final participant has been sent a debrief email/letter. If the pilot trial is deemed to be eliciting too many adverse events, then the University Research Ethics Committee (UREC) Chairperson may terminate it early.

### Recruitment

#### Recruitment of licenced premises

All licenced premises are eligible to participate regardless of whether they function under a premises licence or a club premises certificate.

A minimum of one and maximum of six licenced premises will be recruited via posts on Facebook and Twitter; blogs on scientific forums; local newspaper, magazine and radio advertisements; PI’s presentations at local Pubwatch meetings; targeted emails to licenced premises managers; and word of mouth. Recruitment will work on a first come, first served basis.

Managers of licenced premises that contact the PI with an expression of interest will be sent further details of the study by email or post. If they wish to proceed thereafter, then a meeting will be arranged between the premises manager and the PI. If the PI regards the venue as suitable to host the study, and the premises manager wishes to proceed, both parties will agree on the dates and times for a minimum of four study sessions. During study sessions, the licenced premises will still be open to the public. The licenced premises manager will sign a letter of agreement for research access.

#### Recruitment of participants

Fifty-two participants will be recruited through posts on the licenced premises social media accounts and through placing flyers and posters within the premises.

The recruitment advertisements will ask people who are interested to contact the PI via email or telephone. Once a potential participant has contacted the PI, they will be sent an invitation to participate in the study, a participant information sheet (PIS) and a link to an online eligibility survey. By default, these documents will be sent electronically. The documents will be sent through the post upon request with a stamped addressed envelope.

Recruitment will commence two months before the initial study session at each participating licenced premises.

Recruitment will continue until the sample size (*n* = 52) has been reached.

Recruitment will be monitored by the PI. The PI will stop recruiting at a participating licenced premises if either:

- The manager of the licenced premises expresses they do not wish for any more study sessions to take place (after the agreed four sessions)
- The licenced premises is failing to yield participants at an adequate rate: less than four per study session
- It is no longer feasible for the licenced premises to host the study

The PI may recruit from multiple participating licenced premises at any one time, and this will be at their discretion.

#### Screening

Potential participants will be screened to assess their eligibility by completing an electronic survey using Qualtrics software. The survey will be sent through the post with a stamped addressed envelope upon request.

The PI will analyse the survey responses and contact those who are eligible to arrange two study sessions. Study sessions will be a month apart, on the same day of the week and at the same start time.

Those who do not fulfil the eligibility criteria will be sent an email or letter to thank them for expressing an interest in the study and explaining the reason why they are not eligible.

#### Consent procedure

When potential participants leave their contact details at the end of the electronic screening survey, they consent to their contact details being made available to the research team and for them to be contacted in relation to the study at any given time.

Individuals will take a breathalyser test when they arrive for their first study session and those whose breath alcohol concentration (BrAC) is equal to or below the UK’s drink-drive limit, of ≤ 35 μg/100 ml breath, will be asked to complete a consent form. If the individual’s BrAC exceeds this level, they will be told they cannot take part in the study during the current session as they may be too intoxicated to give informed consent.

Potential participants will be given the time they require to read the consent form (or have it read to them by the PI), ask any questions and complete the form. They will have received the PIS at least 48 h before their study session, so their consent will be regarded as fully informed.

### Allocation

#### Sequence generation

Participants will be randomly assigned to the order that they receive BL and B, using the AB/BA format, therefore counterbalancing conditions. A separate computer-generated randomisation sequence will be produced for each study venue using Randomization.com software [31].

#### Concealment

The first “treatment” label (pink or purple) designated to each subject in the randomisation sequence will be translated as a discrete, coloured label on a randomisation card that will be concealed in a sealed and numbered opaque envelope. The sealed envelopes will be placed in a pile, which will be overturned and placed within a box once all envelopes are present so that the sequence is in ascending numerical order.

#### Implementation

The chief investigator (CI: DF) will generate the allocation sequence and conceal the allocation. The PI will enrol participants and assign them to the interventions by asking them to take the next numbered envelope from the sequence and opening it.

#### Blinding

The participants and the RA will be blinded to the intervention and control products and the order in which they are assigned. Due to limited study personnel, the PI cannot be blinded.

Randomisation cards will display a colour-coded label, and the participants and the RA will be unaware of the colour-coding system. Coloured labels will be placed on the de-identified lager cans that will correspond to the coloured labels on the randomisation cards. The RA will ask the participants to show their randomisation card when they purchase a study-specific drink, and the colour of the label on the card will inform the RA which drink to serve.

#### Emergency un-blinding

In exceptional circumstances, whereby the participant’s welfare would be compromised without the disclosure of the alcohol product that they have consumed, the participant and any other relevant persons will be un-blinded to the intervention and/or control. Participants who are un-blinded will be removed from the study. The PI will report all disclosures to the CI.

### Data collection methods

#### Baseline measures

Participants’ BrACs will be measured at the start of each of their study sessions with an Alcosense Pro Fuel Cell Digital Breathalyser, using a one-way valve mouthpiece. The breathalyser is accurate to − 0.00‰ BrAC, + 0.1‰ BrAC, and will be calibrated annually by Alcosense [32].

#### Data collection methods

The PI will maintain up-to-date records that cover point 1 in the “Outcome measures” section. Feedback from members of the research team, participating licenced premises and participants will be obtained and recorded throughout the study.

Electronic datasets will be created in Excel to monitor:

- Licenced premises that are approached by the PI
- Landlords/managers who express willingness to participate
- Landlords/managers who sign a letter of access
- Participants who consent to participate (and at each separate participating licenced premises)
- Participants who consent and do not complete two study sessions
- Participants who consent and drop out during or after the intervention study session
- Participants who consent and drop out during or after the control study session

To track the number of study-specific drinks served, the RA will stamp the participant’s randomisation card each time they are supplied with a fresh drink. The randomisation card will be handed to the PI at the end of each study session.

Participants will be briefed that if they do not consume the entirety of a study-specific drink they should return their drinking vessel to the RA. The RA will alert the PI who will measure the amount of alcohol that has been left in the vessel. The PI will quantify, in UK units, the amount of alcohol that each participant has left throughout each study session and deduct this from the number of UK units of alcohol served to each participant, which will be calculated by the PI. The number of UK units of alcohol will be converted to, and additionally displayed as, grams of alcohol for an international audience.

The PI will also record the times at which each participant commences and concludes each of their study sessions. This will enable a comparison between the duration of participants’ study sessions under each of the study conditions, which will indicate whether participants may be concluding their study sessions and then consuming non-study drinks.

#### Collecting data from deviant participants

Participants who are seen by the PI, the RA or the BP to be obviously and persistently breaching the protocol will be removed from the study, and their data will not be utilised. Similarly, if a participant wishes to discontinue in the pilot trial during a study session, no further data will be collected.

### Data analysis

The efficacy and efficiency of the study processes and the rates of licenced premises recruitment, participant recruitment and attrition will be analysed and reported using descriptive statistics.

Mean value and 95% confidence intervals will be used to compare the number of UK units of alcohol consumed and the mean duration of participation in study sessions, between the intervention arm and the control arm.

The mean and SD of the number of UK units of alcohol consumed will be used to calculate a sample size for future, larger scale studies. The components of the sample size calculation will be delta (mean difference/SD of mean difference) = derived from a calculation of the data, alpha (sig.level) = 0.05, beta (power) = 0.8.

A *t* test, between the sequences AB and BA, will be undertaken to test for a period effect.

### Monitoring

#### Safety/harms

There is no reason to believe that this study will lead to an excessive number of adverse events. Although participants will be consuming alcohol, they will be briefed to consume alcohol in their normal manner and processes have been put in place to safeguard against study-related harms. Any adverse events that are reported to the PI will be logged using an adverse event form and reported to the CI and the UREC Chairperson. It will be the UREC Chairperson’s decision whether to terminate the study should the number of adverse events reach a level beyond which would be deemed unacceptable.

#### Participant confidentiality and access to data

Data will be de-identified, using a numerical code, where this is feasible. The final anonymised trial dataset will be made publically available in a repository as detailed in the “Data handling, record keeping and retention” section. In accordance with OBU’s Research Data Management Policy, participants will remain anonymous in the thesis and any publication(s) that result(s) from the study [33].

Participants’ information will be used for research purposes only and will only be accessed by the PI, the CI and the co-investigator (Co-I: ED).

#### Data handling, record keeping and retention

Relevant information from electronic correspondence with participants will be encrypted and saved in password-protected folders on an Oxford Brookes University (OBU) computer’s hard drive and deleted from the email account. All hard correspondence will be stored in a locked filing cabinet within a lockable room at OBU.

All hard data collected at the study sites will be securely transferred to locked filing cabinets within a lockable room at OBU at the first available opportunity following each study session. Prior to this, it will be stored in locked cabinet drawers at the PI’s residence. Identifiable and non-identifiable information will be transferred and stored separately.

Qualtrics [34] will host the eligibility surveys, with whom OBU’s Faculty of Health and Life Sciences hold an agreement. Identifiable data from the eligibility surveys will be stored in encrypted and password-protected Excel spreadsheets on an OBU computer’s hard drive.

If a participant withdraws from the study, then all of their information will be destroyed.

Datasets will be kept in accordance with the General Data Protection Regulation (GDPR) [35] and OBU’s Research Data Management Policy [33]. The latter states that study data will be offered and assessed for deposit and retention in a University repository, such as the Research Archive and Digital Asset Repository (RADAR). Data on RADAR will be kept for a minimum of 10 years.

#### Data monitoring and auditing

Progress with data collection will be discussed by the PI, the CI and the Co-I who will meet on a fortnightly basis.

OBU has procedures in place to audit students’ conduct during, and output from, their research. This audit process will accommodate the current study, which is part of a PhD programme.

### Financing and insurance

#### Licenced premises incentives

Each licenced premises that hosts a minimum of four study sessions will be given £500 via an invoice and bank transfer. The licenced premises will also retain all of the participants’ payments for study-specific drinks.

#### Participant incentives

Every participant who completes the trial will be automatically entered into a free prize draw to win one of two prizes of £100, delivered via bank transfer. Participants can opt out of entry into the free prize draw by checking a box on the consent form.

#### Legal liability/insurance

OBU has liability insurance for this research project.

## Discussion

The aim of this pilot trial is to assess the feasibility of a RCT to assess the effect of alcohol strength on alcohol consumption in a single drinking occasion within licenced premises; to our knowledge, no trials to date have investigated this. If the pilot trial is successful, a larger scale RCT could be undertaken, which would test an intervention that has the potential to improve public health by reducing alcohol consumption.

The pilot trial is of a cross-over design, which has the advantages of eliminating between participant variation, and being more economic due to a smaller sample. Whilst there is no risk of a carryover effect, the main disadvantage of using a cross-over design in this study is the potential for a period effect. This will be tested for during data analysis and, if present, subsequently reported.

Whilst one of the main advantages of this study is its naturalistic design, due to the complexity of undertaking research within licenced premises, some compromises have been necessary. For example, ideally the intervention and control lagers would be supplied from “tapped” barrels to align with how the majority of lager is sold to customers within licenced premises. As this is not feasible for a small-scale study, lager will be supplied from duct-taped cans. Although this reduces the face validity of the study, other measures have been put in place to improve the study’s face validity. For instance, lager will not be sold by the can but poured from multiple cans into a pint glass so that a full pint is served.

There are some associated challenges that may be encountered during the pilot trial. These include recruiting licenced premises, retention of participants across two study sessions and participant adherence to the study protocol. Processes that have been put in place to minimise these challenges are discussed below.

### Recruiting licenced premises

A number of licenced premises managers who have been given details of the pilot trial have expressed that their business is tied to a brewery, meaning they are not at liberty to sell any products on the premises that are not provided by the brewery. If a licenced premises is tied to a brewery, then it would be the responsibility of the licenced premises manager to gain permission from the brewery to host the study: to date, no manager has been willing to do this. Therefore, recruitment has focused on “free houses”, which are licenced premises that may obtain stock from any supplier. In particular, free houses that have links to members of the research team, that are involved in local community or charitable projects, or that function as bars within sports clubs have proven particularly fruitful. Such licenced premises are more likely to be incentivised by the financial gain: £500 plus money from the sales of study-specific drinks.

### Retention of participants

The two study sessions that participants are required to attend will be one month apart to control for confounding from day of the month, particularly around payday. To reduce the chance of attrition, participants will be sent reminder emails or letters one week and 24 h before their second study session.

An additional concern is that participants may not like the study-specific drink they have purchased and will withdraw from the pilot trial during a study session. To incentivise participants to remain in the pilot trial, and continue to purchase study-specific drinks only, these drinks will cost approximately one third less than the cheapest lager sold within the venue. An additional incentive of entry into a prize draw to win one of two prizes of £100 has been included to reduce attrition.

### Participant adherence to the study protocol

As participants will be free to mix with other participants and non-participants during their study sessions, there is concern that they can easily obtain and consume drinks other than that which they have been assigned to. Encouraging groups of friends to participate in the study together may reduce the risk of contamination, as everyone within the group will be bound by the same rules and restricted to study-specific drinks. Recruitment advertisements will therefore reflect this strategy. Additionally, participants will be reminded that if they do not adhere to the protocol they will be withdrawn from the study and lose the opportunity to win £100.

As this is a pilot trial, suitable changes will be implemented if any unforeseen problems occur. These will be discussed when assessing the feasibility of a definitive RCT.

## Abbreviations

ABV: Alcohol by volume
B: Becks lager
BL: Bud Light lager
BP: Bar person(s)
BrAC: Breath alcohol concentration
CI: Chief investigator
Co-I: Co-investigator
GDP: Gross Domestic Product
GDPR: General Data Protection Regulation
OBU: Oxford Brookes University
PHRD: Public Health Responsibility Deal
PI: Principal investigator
PIS: Participant information sheet
RA: Research assistant
RADAR: Research Archive and Digital Asset Repository
RCT: Randomised controlled trial
SD: Standard deviation
UK: United Kingdom
UREC: University Research Ethics Committee
